# Sex-related immunity: could Toll-like receptors be the answer in acute inflammatory response?

**DOI:** 10.3389/fimmu.2024.1379754

**Published:** 2024-05-21

**Authors:** Alexandros Popotas, Georges Jacques Casimir, Francis Corazza, Nicolas Lefèvre

**Affiliations:** ^1^ Laboratory of Pediatrics, Université Libre de Bruxelles, Brussels, Belgium; ^2^ Laboratory of Translational Research, Université Libre de Bruxelles, Brussels, Belgium; ^3^ Department of Pulmonology, Allergology and Cystic Fibrosis, Queen Fabiola Childrens University Hospital (Hôpital Universitaire des Enfants Reine Fabiola) – University Hospital of Brussels (Hôpital Universitaire de Bruxelles), Brussels, Belgium; ^4^ Laboratory of Immunology, Centre Hospitalier Universitaire (CHU) Brugmann, Université Libre de Bruxelles, Brussels, Belgium

**Keywords:** TLR-Toll-like receptor, innate immunity, sex, X chromosome, hormones, sepsis, infection, trauma

## Abstract

An increasing number of studies have highlighted the existence of a sex-specific immune response, wherein men experience a worse prognosis in cases of acute inflammatory diseases. Initially, this sex-dependent inflammatory response was attributed to the influence of sex hormones. However, a growing body of evidence has shifted the focus toward the influence of chromosomes rather than sex hormones in shaping these inflammatory sex disparities. Notably, certain pattern recognition receptors, such as Toll-like receptors (TLRs), and their associated immune pathways have been implicated in driving the sex-specific immune response. These receptors are encoded by genes located on the X chromosome. TLRs are pivotal components of the innate immune system, playing crucial roles in responding to infectious diseases, including bacterial and viral pathogens, as well as trauma-related conditions. Importantly, the TLR-mediated inflammatory responses, as indicated by the production of specific proteins and cytokines, exhibit discernible sex-dependent patterns. In this review, we delve into the subject of sex bias in TLR activation and explore its clinical implications relatively to both the X chromosome and the hormonal environment. The overarching objective is to enhance our understanding of the fundamental mechanisms underlying these sex differences.

## 1 Introduction

Sex-dependent inflammatory responses are well established in the realm of adaptive immunity (1, 2). However, in the critical hours immediately following cellular insult, the rapid and robust inflammatory reaction primarily relies on innate immunity, encompassing various aspects of activation and regulation. Numerous examples illustrate the importance of innate immunity, as the breakdown of one of its pathways can lead to severe clinical consequences (3). Epidemiological evidence now corroborates disparities between men and women in the early immune response, with sex emerging as a distinct prognostic factor in acute inflammation (1). Sex-specific responses are observed in inflammatory conditions such as acute infections, trauma, or surgery. The recent coronavirus disease 2019 (COVID-19) pandemic has further highlighted these sex differences (4–7). Nevertheless, the precise mechanistic underpinnings of this sexual dimorphism remain elusive. While hormonal influence was initially attributed as the main driver, emerging evidence suggests the involvement of an alternative mechanism, potentially linked to genes residing on the sex chromosomes that play a role in the inflammatory response (8). Among the various mediators, Toll-like receptors (TLRs), which are specific receptors of the innate immune system, play a pivotal role in activating inflammatory markers associated with sex-related inflammatory responses (9). In this review, we shed light on the sex bias in TLR expression and activation, striving to elucidate the underlying mechanisms, including the influence of the hormonal environment and contributions from the X chromosome.

## 2 Sex differences in acute inflammatory responses

### 2.1 Infectious diseases

#### 2.1.1 Bacterial infections

Substantial disparities in the incidence, prognosis, and immune response between men and women become evident in the context of acute infectious diseases. Clinical studies have consistently demonstrated a higher incidence of bacteremic infections among men, including conditions such as invasive pneumococcal disease (10), *Pseudomonas aeruginosa* (11), or *Staphylococcus aureus* (12) infections. When these infections progress toward sepsis, the male sex emerges as a significant risk factor, as higher mortality and sequelae are reported in men (12–15). In a large cohort including more than 10 million cases of sepsis, the male sex was associated with a higher incidence (16). Similarly, men admitted to intensive care units (ICUs) exhibit a heightened incidence of sepsis and shock (17). Despite these findings suggesting that the male sex is linked to an elevated risk of bacteremic infections, it is important to note that the incidence of *Escherichia coli* bacteremia is higher in women (18, 19). This observation, however, can be attributed to anatomical differences of the urinary tract, where *E. coli* urinary tract infections (UTI) predominate in women. Nevertheless, men face a greater risk of morbidity (20, 21) and mortality (22) from the complications of UTIs and require more extended treatment to eliminate the bacterial infection (23).

Sex differences are also evident in bacterial lung infections. The male sex is a risk factor in the context of community-acquired pneumonia (CAP) (24–27). Men not only experience a more unfavorable disease progression, but also exhibit a higher rate of admissions to ICUs (26, 27). The most common pathogen isolated in CAP is *Streptococcus pneumoniae* (28), with men being disproportionately affected by severe pneumococcal CAP (29). Tuberculosis, another prominent bacterial infection with a global impact on patient mortality, also displays sex-based disparities. *Mycobacterium tuberculosis* primarily afflicts men, who concurrently manifest elevated mortality rates compared with women (30, 31).

These clinical observations align with corresponding biological findings observed in sepsis and other infectious diseases. An investigation involving prepubertal children admitted to ICUs for sepsis revealed higher maximum white blood cell and neutrophil counts, lower pH, and higher C-reactive protein (CRP) levels in women (32). In the early stages of the disease, general biological markers of inflammation are more elevated in women, indicating a heightened activation of innate defense mechanisms. However, over time, a distinct kinetic pattern emerges, highlighting differences in the return to homeostasis between women and men. In contrast to these general inflammatory markers, levels of proinflammatory cytokines such as interleukin 1β (IL-1β), IL-6, and tumor necrosis factor alpha (TNF-α) are higher in men, whereas women exhibit elevated levels of anti-inflammatory mediators, notably IL-10 (13, 33). Men with CAP, who face a worse prognostic compared with women with CAP, present stronger release of TNF-α (34).

Findings from studies involving human subjects are congruent with observations in animal models. In an experimental model of sepsis induced by cecal ligation, female mice exhibit higher survival rates and maintain splenocyte functions (35), whereas male mice release higher levels of IL-6, TNF-α, and prostaglandin E2 (PGE2) (36). Among mice, there is a heightened incidence of systemic pneumococcal infection and pneumococcal respiratory infection in males, who also present higher mortality rates than their female counterparts (37). Further, male mice infected with *S. pneumoniae* produce significantly greater quantities of proinflammatory cytokines, including IL-6, IL-12p70, interferon gamma (IFN-γ), granulocyte colony-stimulating factor (G-CSF), and IL-17A (37). *Mycobacterium bovis*, which causes bovine tuberculosis, and *Mycoplasma pulmonis*, responsible for respiratory mycoplasmosis in mice, both display a more severe clinical outcome and higher mortality in males (31). Interestingly, *M. pulmonis* induces alveolar neutrophil infiltrate with edema and hemorrhage in males, resembling an acute alveolar inflammatory response. Conversely, female lungs with respiratory mycoplasmosis display macrophage infiltration with minimal neutrophils, akin to a chronic peribronchial inflammatory response (31).

Sex disparities in bacterial infections extend beyond mammals, with documented differences in defense against pathogens observed in insects and birds (38, 39).

#### 2.1.2 Viral infections

Sexual dimorphism in clinical outcomes and immune responses is notably evident in viral infections. Throughout the COVID-19 pandemic, epidemiological reports have consistently described a better clinical course in women (4–7), with men presenting higher levels of proinflammatory cytokines (40). Similarly, during the outbreaks of severe acute respiratory syndrome (SARS-CoV) and Middle East respiratory syndrome (MERS-CoV), men showed a higher incidence and more severe disease (41, 42). Sex disparities are also apparent in *Paramyxoviridae* infections; boys infected with respiratory syncytial virus (RSV) or human metapneumovirus (hMPV) develop more severe clinical manifestations compared with girls (43–45).

Dengue virus (DENV) a substantial global health concern, also displays sex differences, with men experiencing a disadvantage (46).

In contrast to the aforementioned viruses, among adults infected with influenza A, women are more susceptible to an increased risk of severe clinical presentations (47), accompanied by higher IFN-γ levels compared with men (44, 48, 49).

Sex-specific disparities in viral responses have been corroborated in animal models. Male golden Syrian hamster exhibit a poorer outcome and develop more severe SARS-CoV-2 pneumonia than their female counterparts (50) and male mice infected with RSV display impaired IFN-β expression and viral control (51). Female mice infected with equal viral loads of influenza virus to males, experience a worse outcome, with greater cytokine and chemokine release in their lungs (44, 52).

### 2.2 Trauma and surgery

#### 2.2.1 Trauma

Sex-specific patterns in complication rates are evident among trauma patients. As reported by Mörs et al. (53), men exhibit higher injury scores and an increased risk of developing sepsis following trauma. In polytraumatized patients, men have a higher incidence of multiple organ dysfunction syndrome and sepsis (54). Offner et al. (55) further emphasized that the male sex constitutes a significant risk factor for major post-trauma infections. Moreover, in cohorts involving trauma patients with either blunt or penetrating injuries, post-trauma complications such as pneumonia (56, 57), sepsis (58), and multiple organ failure (59) are more prevalent in men. Factors such as the length of ICU stay and ventilator use are influenced by the patient’s age and the severity of the trauma, but they predominantly impact men (60).

While disparities in trauma complications between men and women are well established, the impact of sex on survival after injury remains less clear. Mortality rates stemming from penetrating or blunt trauma, as well as post-trauma infection, exhibit a pronounced male predilection (60–63). While studies delineating mortality disparities across age cohorts manifest certain inconsistencies, discernible trends persist, albeit with variation across investigations (60, 62, 63).

Biological sex differences become apparent in trauma patients, with men producing significantly higher IL-6 levels during the initial 2 days after injury (53). Similar findings have been reported in polytraumatized patients, where men exhibit higher levels of IL-6 and IL-8 compared with women (54). Additionally, men with multiple injuries and those who develop post-trauma sepsis produce increased levels of IL-6, IL-8, and TNF-α (64).

Animal studies further emphasize the existence of inflammatory sexual dimorphism in the context of trauma, with varying survival rates in different species and trauma contexts, emphasizing the complexity of sex-related responses to trauma (65, 66). In experimental mouse trauma models, male macrophages produce higher levels of IL-6 (67, 68).

Burn injuries are another type of trauma with discrepant results regarding complications and survival in humans. Several authors have demonstrated increased length of hospital or ICU stays and higher mortality rates in women (69–72). Cumming et al. (73) identified an association between the male sex and the development of multiple organ dysfunction and severe sepsis following burn trauma. The prognosis may be influenced by the duration of the process, with worse outcomes in men during the acute phase, while chronic inflammation consequences may exacerbate the prognosis in women.

The conflicting outcomes observed in burn trauma studies may stem from variations in the timing of analyses and the involvement of cofactors such as skin loss, fluid depletion, hypovolemic shock, and secondary infections. Additionally, the inconsistencies may arise from the heterogeneous nature of the aggression, incorporating elements like fractures, hematomas, bacterial translocations. Consequently, the comparison between study populations becomes notably challenging.

#### 2.2.2 Surgery

Surgery itself acts as an inflammatory trigger, yet there is a scarcity of research in this domain. Similarly to the studies on trauma, the presence of confounding factors such as underlying pathology or infectious complications adds complexity to research endeavors in surgery and inflammation.

Among patients who have undergone surgery, men present a higher mortality risk and are more susceptible to complications. In a study involving 512 surgical patients, men demonstrated a higher risk of developing major respiratory complication, with no sex-specific differences regarding the duration of the operation, the length of hospital stay, or mortality rates (74). Similarly, in a study on surgical intensive care patients, the authors observed a higher incidence of severe sepsis or septic shock among men (17), and a large retrospective analysis revealed a higher perioperative morbidity in male surgical patients (75).

Sex-specific cytokine profiles have been identified in surgical patients, revealing an excessive release of TNF-α and diminished levels of IFN-γ in stimulated male peripheral blood mononuclear cells (PBMCs) (76).

There is very limited information on surgery-induced inflammation and mortality in animals. In equine surgical operations for colic, some researchers have reported higher mortality rates for males (77), while others studies have not confirmed these results (78).

## 3 TLRs

### 3.1 TLR structure

Whether in humans or animals, the early inflammatory response to infection or injury is predominantly orchestrated by the innate immune system. This crucial defense mechanism identifies pathogens or damaged cells through pattern recognition receptors (PRRs). Among the well-studied PRRs, TLRs stand out. They rapidly recognize infectious agents or damaged particles, subsequently initiating signals for the elimination of microbial pathogens or the activation of adaptive immune responses.

The human innate immune system comprises 10 TLRs, each characterized by a distinct structural composition. These receptors consist of two essential domains. The N-terminal end features an extracellular leucine-rich repeat (LRR) domain that functions as a ligand binder. Conversely, the C-terminal end contains the intracellular domain known as the IL-1 receptor (Toll/IL-1 receptor [TIR]), responsible for initiating the signaling cascade (79, 80).

TLRs are expressed in diverse cellular locations; some are found on the cell membrane, while others are located in endosomes within the cell’s cytoplasm. Specifically, TLR3, TLR7, TLR8, and TLR9 are endosomal, whereas the remaining TLRs mainly function as membrane-bound receptors (79, 80). These TLRs are expressed in a wide array of cell types, including but not limited to endothelial cells; epithelial cells; parenchymal cells; synovial fibroblasts; and hematopoietic-derived cells such as dendritic cells (DCs), macrophages, neutrophils, B cells, and T cells (79, 80).

### 3.2 TLR signaling

The extracellular N-terminus of TLRs is responsible for ligand binding, which varies depending on the specific type of TLR. After ligand recognition, signaling cascades are enhanced through the intracellular C-terminal domain, leading to the activation of transcription factors and the subsequent induction of cytokine expression (9, 80, 81).

**Figure 1:**
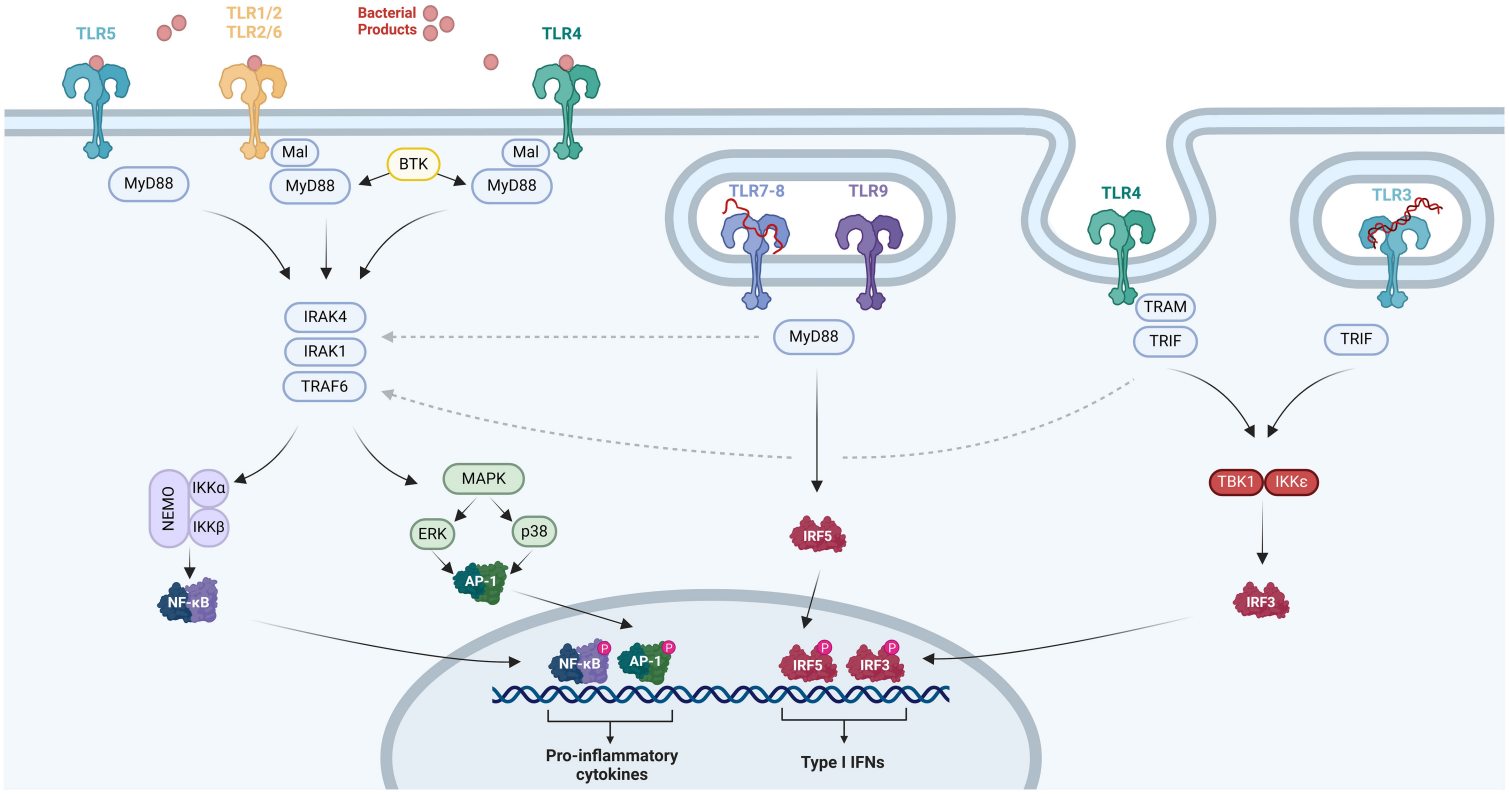
TLR pathways. The TIR domain within TLR2, TLR4, or TLR5 interacts with MyD88, initiating the activation of IRAK1, IRAK4, and TRAF6. This interaction triggers the phosphorylation of the NF-κB complex and instigates NF-κB-regulated factors. Additionally, it activates p38 MAPK and ERK1/2, which subsequently induce AP-1 activity. The activation of NF-κB and AP-1 prompts the production of proinflammatory cytokines. TRIF functions as an adaptor molecule for TLR3 and TLR4, facilitating NF-κB activation through its interaction with TRAF6, while also initiating TBK1 and IKKϵ activation. TBK1 and IKKϵ phosphorylate IRF3, leading to the expression of IFN-a/b. TLR7, TLR8, and TLR9 are implicated in IFN expression through TRIF-independent mechanisms, potentially involving IRF-5 or the MyD88–IRAK1–TRAF6 pathway, although the exact mechanism remains to be elucidated. *Created with BioRender.com*.

The intracellular domain of TLRs, known as the TIR domain, is essential for mediating signal transduction. This domain interacts with various adaptor proteins, including myeloid differentiation factor 88 (MyD88), MyD88-adapter-like (Mal), TIR domain-containing adaptor inducing IFN-β (TRIF), and TRIF-related adaptor molecule (TRAM) (9, 80–83). MyD88 is a common adaptor utilized by all TLRs, with TLR3 being a potential exception, whereas the other adaptor proteins are much more restricted in their TLR interactions. Mal is involved in signaling via TLR2 and TLR4, TRIF is a component of the signaling machinery for TLR3 and TLR4, and TRAM is specifically associated with TLR4 (**Figure 1**) (9, 80–83).

Upon activation of these adaptor proteins, members of the IL-1 receptor-associated kinase (IRAK) family, including IRAK1 and IRAK4, as well as TNF receptor-associated factor 6 (TRAF6) and inhibitors of nuclear factor kappa-light-chain-enhancer of activated B cells (NF-κB) kinases (IKKs), facilitate signal transmission. This activation results in the phosphorylation of the NF-κB complex, leading to the activation of NF-κB-regulated factors and, concurrently, phosphorylation of mitogen-activated protein kinases (MAPKs), including p38 MAPK and extracellular signal-regulated kinase 1/2 (ERK1/2), which induce activator protein 1 (AP-1) activation (9, 80). These transcription factors are primarily responsible for the release of proinflammatory molecules (84). Additionally, Bruton’s tyrosine kinase (BTK) can contribute to this pathway through its interactions with MyD88 and IRAK1 (**Figure 1**) (9, 80–83). It is noteworthy that *BTK*, *IRAK1*, and *IKBKG*—which encodes IKKγ, also known as NF-κB essential modulator (NEMO)—are encoded on the X chromosome at Xq21 and Xq28, respectively (85).

TRIF can directly activate NF-κB through interaction with TRAF6 and is also capable of initiating signal transduction to TRAF family member associated NF-κB activator binding kinase (TBK) 1 and IKKϵ. These kinases phosphorylate and activate IFN regulatory factor 3 (IRF3), which is essential for the induction of IFN-α/β. Therefore, TRIF plays a central role as the adaptor molecule enabling TLR3 and TLR4 to induce the expression of IFN-α/β (**Figure 1**) (9, 80–82). In contrast, TLR7, TLR8, and TLR9 appear to trigger IFN expression through TRIF-independent mechanisms, although the precise mechanism remains unclear. Some data suggest the involvement of IRF-5 or the MyD88–IRAK1–TRAF6 pathway (**Figure 1**) (9, 80–82).

The TLR4 and TLR2 pathways are two well-known pathways of the innate immune system. TLR2 usually builds heterodimer structures with TLR1 or TLR6, while homodimerization is observed for TLR4. TLR4 plays a critical role in the inflammatory response to gram-negative bacteria through recognition of lipopolysaccharide (LPS). To achieve this, TLR4 associates with the CD14 receptor and the MD-2 co-receptor, allowing for the recognition of LPS and the initiation of downstream signaling (82).

On the other hand, TLR1–TLR2 detects pathogens containing molecules of triacylated lipoproteins and gram-negative bacteria (9, 79–81, 86). Meanwhile, TLR2–TLR6 is responsible for recognizing microbes with diacylated lipoproteins, zymosan in fungi, bacterial lipoteichoic acid as seen in *Staphylococcus* spp., peptidoglycans in gram-positive bacteria, and released microbial heat shock proteins (HSPs) (9, 79–81, 86).

TLR3 recognizes double-stranded RNA (dsRNA) viruses and triggers antiviral immune responses by promoting the secretion of proinflammatory cytokines and type I IFN. On the other hand, TLR7 and TLR8 are specialized in detecting viral single-stranded RNA (ssRNA). TLR9 recognizes unmethylated CpG DNA motifs belonging to bacteria, fungi, viruses, and protozoa (79, 86).

In addition to infectious ligands, cellular damage from pathogens or injuries, leads to the release of molecules known as damage-associated molecular patterns (DAMPs). These DAMPs can contribute to inflammation and potentially exacerbate tissue damage or impair wound healing (87, 88). Normally confined within the intracellular compartment, DAMPs are inadvertently released into the extracellular environment following cellular damage (87, 88). Examples of DAMPs include RNA, DNA, high-mobility group box-1 protein (HMGB-1) associated with chromatin, adenosine triphosphate (ATP), degradation products like uric acid, and stress-induced molecules such as HSPs (87, 88).

TLRs have the ability to recognize DAMPs and to elicit an appropriate and fast response. Several DAMPs have been shown to induce cytokine release through the recognition of TLR2 and TLR4. Furthermore, β-defensin-2 and β-defensin-3, as danger molecules, activate TLR2 and TLR4 (89, 90). TLR1 also plays a role in recognizing β-defensin-3 (87, 88). Nucleic acids are recognized by TLR3, TLR7, and TLR8, which are activated by messenger RNA (mRNA), ssRNA, and IgG-chromatin complexes, respectively (87, 88).

### 3.3 The implication of TLRs in acute inflammatory diseases

#### 3.3.1 TLRs and bacterial infections

TLR2 and TLR4 play a significant role in the recognition of these pathogens and the subsequent release of inflammatory cytokines during sepsis (84, 91, 92). The presence of a specific IRAK-1 variant haplotype, a crucial protein in the TLR2 and TLR4 pathway, is linked to increased nuclear translocation of NF-κB, contributing to a worse prognostic clinical presentation in sepsis (93). Moreover, TLR2 and TLR4 polymorphisms are associated with a higher risk of staphylococcal or gram-negative infections, further underscoring the involvement of TLRs in sepsis (94, 95). *TLR2* and *TLR4* mRNA upregulation is also described in monocytes from patients with sepsis (96, 97).

Multiple TLRs recognize *S. pneumoniae*, the most frequent bacteria in CAP: TLR2 recognizes lipoteichoic acid and lipoproteins from the pneumococcal cell wall (91), TLR4 detects pneumolysin (91, 98) and TLR9 is activated through recognition of pneumococcal bacterial DNA which contains unmethylated CpG motifs (89). All three of these receptors regulate the NF-κB pathway to enhance proinflammatory cytokine release (91).

Animal experiments further support the involvement of TLRs in sepsis and pneumococcal lung infection: mice with experimental sepsis exhibit a cytokine pattern dependent on NF-κB and MAPKs (37) and TLR2 and TLR4 knockout (KO) mice display less effective cytokine release compared with wild mice (90).

In murine models of sepsis, TLR2 and TLR4 are upregulated in hepatic and splenic macrophages (90). Similarly, mice with peritonitis show higher expression of *TLR2* and *TLR4* mRNA in the lungs and liver (99). The administration of endotoxin leads to the upregulation of *TLR2* mRNA through TLR4- and MyD88-dependent signaling in alveolar macrophages and endothelial cells (100, 101). This crosstalk between TLR2 and TLR4 is achieved through activated polymorphonuclear neutrophils (PMNs), making their presence a determinant element in the response within TLR pathway (100, 101).

In mice with endothelial cells lacking TLR4, pulmonary neutrophil recruitment is significantly reduced after exposure to LPS, illustrating the importance of alveolar endothelial TLR4 expression in pulmonary neutrophil recruitment during sepsis (102).

In animal experiments simulating pneumococcal lung infection, TLR4 KO mice demonstrate a less vigorous immune response (103, 104). TLR2-deficient mice show decreased release of proinflammatory cytokines (105, 106), and TLR9-deficient mice exhibit weaker lung bacterial clearance compared with wild-type mice (89).

#### 3.3.2 TLRs and viral infections

The role of TLRs in viral infections has gained significant attention in the context of the COVID-19 pandemic. Initially, information about TLR recognition of SARS-CoV-2 was extrapolated from data on SARS-CoV-1. Among the TLRs described in SARS-CoV-1 infection, TLR3 and TLR7 are the most prominent, leading to type I IFN expression through the TRIF–IRF3 pathway (83, 107, 108). There is also evidence for MyD88-dependent pathway activation by SARS-CoV-1, such as NF-κB (109, 110). Furthermore, AP-1 activation has been previously described solely in the context of SARS-CoV infections, with the spike and nucleocapsid proteins of the virus identified as the triggering factors (111, 112).

In the context of severe COVID-19 cases in adults involving a massive release of proinflammatory cytokines, numerous studies have emphasized the role of TLRs. Molecular docking investigations have revealed a strong interaction between TLR4 and the spike glycoprotein of SARS-CoV-2. Additionally, the upregulation of TLR4 and its associated pathway components (CD14, MyD88, Mal, TRAF6, IRAK1, and TRIF) have been observed in PBMCs from patients with COVID-19 (113–115). TLR2 can recognize SARS-CoV-2 by interacting with the SARS-CoV-2 E protein; this interaction amplifies intracellular immune pathways, leading to the activation of an immune response against the pathogen (116).

We previously described the relation between endothelial TLR4 and neutrophil recruitment in experimental models simulating bacterial infection (102). An intriguing observation is that COVID-19 in adults is also marked by a swift and substantial influx of neutrophils (117). This is further reflected in the elevated peripheral blood neutrophil count in patients with COVID-19, with the neutrophil-to-lymphocyte ratio (NLR) serving as an indicator of disease severity (40, 117). Nevertheless, the impact of TLRs and sex on neutrophil levels in COVID-19 remains an understudied area (40). Only one study suggests a male predominance in the NLR among patients with COVID-19, which is associated with a more unfavorable prognosis (118).

Animal models have produced similar findings regarding the implication of TLRs in *Coronaviridae* detection. For SARS-CoV-1, direct evidence points to the implication of TLR4, as TLR4 KO mice are more susceptible to SARS-CoV-1 than wild-type mice, exhibiting higher viral titers (115).

Another virus notorious for eliciting a robust inflammatory response, as well as notable and swift neutrophil recruitment, is RSV (117, 119). RSV activates the NF-κB pathway through TLR2 and TLR4, similarly to bacterial pathogens, to trigger cytokine release and to recruit immune cells into the airways (43). Notably, TLR4 polymorphisms are associated with severe RSV clinical presentations in children (120–123). RSV upregulates TLR4 in airway epithelial cells and monocytes among children with acute bronchiolitis (124, 125). *In vitro* studies on human peripheral macrophages suggest that TLR4 recognizes the RSV F protein (126), and bronchoalveolar lavage analyses have demonstrated elevated neutrophil levels in children with severe bronchiolitis (127–129). Girls with viral bronchiolitis tend to have higher levels of circulating neutrophils compared with boys (130).

Studies in mice underscore the contributions of TLR2 and TLR4 to RSV recognition, with TLR2 KO mice showing a higher viral load and impaired cytokine mediation (131) and TLR4 KO mice displaying an impaired immune response and reduced virus clearance (132). TLR4 also plays a role in recognizing human metapneumovirus and enhancing the innate immune response against this pathogen (133).

Human cytomegalovirus (HCMV) is detected by both membranous and endosomal TLRs. Glycoproteins on the HCMV surface interact with TLR2 and TLR4, resulting in the activation of the NF-κB pathway and subsequent production of proinflammatory cytokines (134). Furthermore, HCMV nucleic acids are recognized by endosomal TLR3, leading to the release of IFN-α (134). Studies investigating mutations or polymorphisms in TLR3 and TLR9 have indicated an increased risk of CMV infection, underscoring the significant role played by these receptors in the recognition of HCMV (134).

The recognition of the DENV involves activation of both the MyD88 and IRF-3 or IRF-5 pathways, dependent on the specific viral components engaged in the interaction with the host’s immune response (135). Specifically, DENV2 triggers TLR3, TLR7, and TLR8, resulting in a robust induction of IL-8 and IFN-α (136, 137). Additionally, the DENV NS1 protein induces signaling pathways associated with TLR2, TLR6, and TLR4, leading to subsequent proinflammatory cytokine production (138, 139). Individuals with TLR4 polymorphisms exhibit an increased susceptibility to DENV infection (140) and patients with dengue hemorrhagic fever demonstrate elevated TLR2 expression in comparison to those with dengue fever (141). In animal models of experimental dengue infection, mice lacking TLR6 exhibit improved survival rates following exposure to DENV2 and NS1, in contrast to wild-type mice (139). In macaques, dengue virus recognition is described through TLR3, TLR7, and TLR8 (142).

As mentioned above, influenza A exhibits a different pattern, with women being more susceptible to an increased risk of severe clinical presentations (47) and higher IFN-γ levels compared with men (44, 48, 49). The influenza virus primarily activates TLR7 and the IRF3 pathway (143) rather than the TLR4 or MyD88-dependent pathways. In rodent models of influenza infection, TRL3- and TLR7-deficient mice are unable to control viral replication and succumb to the disease (144). TLR4 contribution was not demonstrated in TLR4 KO mice with influenza infection, thereby affirming the predominant involvement of TLR3 and TLR7 activation in response to influenza virus (132).

#### 3.3.3 TLRs and surgery or trauma

There are no studies focusing on the impact of surgical procedures on the activation of TLR pathways. In many surgical pathologies, TLRs are already stimulated by pathogens or DAMPs (145). As in humans, the literature available on the interaction between surgery and TLR responses in animals is limited. However, a notable finding comes from experiments involving mice subjected to sternotomy and treated with a TLR9 antagonist. In this context, a decrease in IL-6 production is observed, highlighting the potential significance of the TLR9 pathway in modulating the inflammatory response elicited by surgical procedures (146).

The precise involvement of TLRs in trauma remains a topic of ongoing investigation, with conflicting findings in the literature. Trauma patients with an ISS (injury severity score) over 12 display heightened expression of TLR2 and TLR4 on monocytes (147). However, in patients with an ISS exceeding 25, TLR4 expression on monocytes decreases, and TLR2 expression is similar to healthy subjects (148). Some studies report unaltered TLR4 but reduced TLR2 expression in patients with an ISS greater than 21 (149). These findings are based on limited populations, emphasizing the need to expand these studies before drawing definitive conclusions. Furthermore, thoughtful consideration should be given to the potential limitation in TLR2 and TLR4 availability resulting from their excessive engagement in the context of trauma.

Trauma patients exhibit higher serum levels of IL-6 and IL-10 (147). Previous studies have established a discernible correlation between IL-6 levels and ISS within the initial 72 hours of hospitalization (150). TLR2, -4 and -9 activation results in trauma patients results in an impaired proinflammatory response, as evidenced by lower TNF and IL-6 levels (148, 149) and increased release of IL-10 (148).

Findings from a murine model indicate that TLR4 plays a crucial role in neutrophil accumulation and well as increased TNF-α production (denoted by increased mRNA and protein levels) following experimental hemorrhage (151). Hemorrhage in mice leads to increased upregulation of p38, ERK1/2, and c-Jun N-terminal kinase (JNK) in Kupffer cells, as described by Thobe et al. (152). Furthermore, the release of proinflammatory cytokines mediated by TLR2 and TLR4 is modulated through the p38, ERK1/2, and JNK pathways (152). The inflammatory response after lung injury in mice appears to be TLR9-mediated, as TLR9-deficient mice exhibit reduced proinflammatory cytokine release (153). TLR9-dependent TNF-α and IL-6 production has been shown to rely on JNK (152).

TLRs have also been implicated in burn injuries: The levels of TLR2, TLR4, and TLR9 on DCs are higher in burn patients compared with healthy volunteers. Additionally, TLR2 protein expression is associated with the survival prospects of individuals affected by burn injuries (154). However, in the specific context of burn trauma, hemodynamic and infectious cofactors impede a thorough analysis of the specific TLR implication in thermal injuries. As highlighted in the above studies on TLR analysis in trauma patients, the constrained sample size underscores the need for further investigations in this field.

In murine models, TLR function is impaired in the context of burn injuries. Macrophages from burned mice show reduced expression of *TLR3* and *TLR9* mRNA compared with macrophages from their healthy counterparts (155). DCs from burned mice have lower *TLR9* mRNA expression, resulting in an altered cytokine release profile characterized by an anti-inflammatory response, including elevated IL-10 levels and reduced production of IL-6, TNF-α, and IL-12p70 (156).

## 4 Sex differences and TLRs

### 4.1 Sex bias in TLRs and their pathways

**Figure 2:**
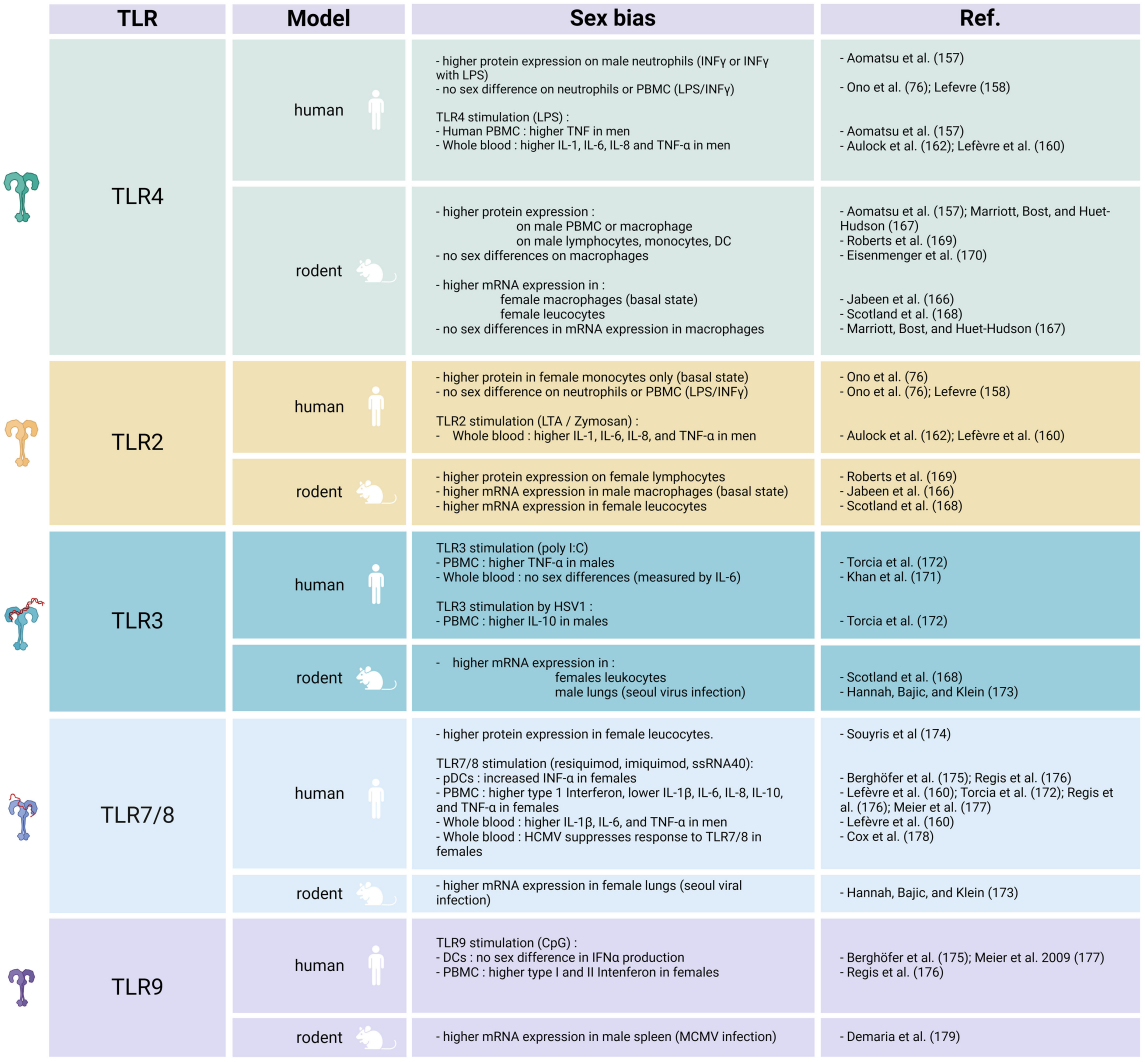
TLR sex differences in humans and rodents. *Created with BioRender.com*.

As discussed above, the literature emphasizes sex-based disparities in the inflammatory response during bacterial, viral infections, and even trauma. What is particularly remarkable is the involvement of TLR pathways in these mechanisms (**Figure 2**), but, surprisingly, very few studies have focused on the relationship between sex and TLR expression within the immune system.

#### 4.1.1 TLR2 and TLR4

There have been diverse results regarding TLR2 and TLR4 expression. An *in vitro* study on neutrophils from healthy young adults stimulated with either LPS or IFN-γ revealed higher TLR4 expression in men (157). Conversely, higher TLR2 expression is reported in monocytes from healthy women (76). However, other researchers have not identified any sex difference in TLR4 or TLR2 expression on human immune cells after exposure to LPS (76, 158).

In some studies exploring the potential influence of sex on TLR responses, researchers have analyzed cytokine release subsequent to TLR activation. *In vitro* stimulation of TLR4 and TLR2 by LPS or zymosan, respectively, results in augmented proinflammatory cytokine release in whole blood or isolated leukocyte populations from men (157, 159–162). Furthermore, after LPS stimulation, men exhibit a higher percentage of IL-12-, IL-1β-, and TNF-α-producing monocytes compared with women (163).

As discussed previously, men tend to produce predominantly proinflammatory cytokines, whereas women primarily release cytokines from the IL-10 family. This distinctive sex-related pattern is further corroborated through TLR2 and TLR4 activation. When subject to whole blood endotoxin stimulation, women demonstrate elevated levels of IL-10, relative to the monocyte concentration, enhancing their ability to regulate the immune response effectively (164). Indeed, IL-10 plays an essential role in maintaining the integrity and homeostasis of the cellular environment during inflammation (165).

Regarding the downstream proteins within the TLR2–TLR4 pathway, male neutrophils present greater activation of ERK, p38, JNK, and Akt upon LPS stimulation. However, there are no discernible sex differences in either IκBα phosphorylation or degradation subsequent to endotoxin exposure (157).

The above studies reveal divergent patterns in the sex-related expression of TLR2 and TLR4 in human. However, activation through ligands primarily elicits an enhanced response in men, with increased cytokine release and higher TLR2- and TLR4-associated proteins. These differences appear to stem from the core pathway, specifically involving the proteins associated with TLRs and the mechanisms governing cytokine release, rather than the receptors themselves.

We previously described the relation between endothelial TLR4 and neutrophil recruitment in bacterial infection (102) or viral infections characterized by a significant recruitment of neutrophils, such as COVID-19 (117) and RSV bronchiolitis (43, 117, 119). The predominant function of TLR2 and TLR4 in men offers a promising avenue of research to identify the etiology of worse clinical outcomes and heightened inflammatory responses observed in men.

Research on TLR2 and TLR4 mRNA and protein expression in animal models has yielded inconsistent results. At basal state, male mice express higher levels of TLR2, while higher TLR4 levels are registered on macrophages from female mice (166). Conversely, certain studies suggest that while there are no discernible sex differences in *TLR4* mRNA expression under baseline conditions, male murine macrophages exhibit higher levels of TLR4 protein on their cell surfaces (167).

These contradictory results also extend to stimulated conditions in animals. Stimulated female rodent peritoneal leukocytes show increased *TLR2* mRNA and *TLR4* mRNA levels (168). However, a communication by Marriot et al. (167) suggests that stimulated murine macrophages do not exhibit a sex difference in *TLR4* mRNA expression. In an infectious model involving rodents infected with coxsackievirus, female mice demonstrate improved survival rates, which correspond to higher TLR2 expression on lymphocytes (169). Females also display lower TLR4 expression on lymphocytes, monocytes, and DCs (169). In the same model, male mice display an increase in TLR2 expression on monocytes and DCs (169). Furthermore, after trauma and hemorrhagic shock, and also following LPS stimulation, mouse macrophages do not display sex-dependent TLR4 expression on their surface (170).

Interestingly, when analyzing cytokine response to LPS stimulation, male subjects display elevated IL-6 levels (167, 168). Moreover, male-derived macrophages exposed to LPS exhibit higher levels of IL-1β (167). Once again, similarly to human studies, it appears that sex differences are more evident within the context of the TLR2 and TLR4 pathways than in the receptors themselves.

#### 4.1.2 TLR3

Limited information is available regarding the TLR3 sex bias in infectious diseases. In human studies, researchers have assessed activation of the TLR3 pathway by measuring cytokine release after stimulating PBMCs with polyinosinic:polycytidylic acid (poly I:C). They have registered significant sex differences only for TNF-α, specifically higher levels in male PBMCs (171, 172). Stimulation with herpes simplex virus 1 (HSV1), a double-stranded DNA virus, induces higher IL-10 production in men, although it is essential to note that this virus also engages TLR9 (172).

Findings from animal experiments indicate greater *TLR3* mRNA expression in peritoneal leukocytes from female mice, although the authors did not investigate protein expression under infectious conditions (168). In virus challenge models involving Seoul virus, female rats exhibit lower TLR3 expression compared with male rats (173).

#### 4.1.3 TLR7 and TLR8

Higher expression of TLR7 has been registered in women (174). As described above, TLR7 activates the type I IFN signaling pathway. Consequently, increased levels of type I IFN are observed in immune cells from adult and adolescent women following exposure to TLR7 ligands (175, 176). In patients with COVID-19, women demonstrate elevated production of IFN-α2 (2).

Stimulation of TLR7 and TLR8 leads to amplified production of both type I and type II IFNs and decreased levels of IL-1β, IL-6, IL-8, IL-10, and TNF-α in female PBMCs (160, 172, 176, 177). In whole blood samples, resiquimod induces significant sex differences only in IL-1β, IL-6, and TNF-α, with higher production in men (160). Interestingly, HCMV suppresses the response to TLR7/8 stimulation specifically in women (178). In rats, greater TLR7 expression occurs in females following viral exposure (173).

Certain viruses, such as influenza mainly activates IRF3 pathway through TLR7 triggering (143). This leads to a distinct pattern where women exhibit greater susceptibility to an increased risk of severe clinical presentations (47) and elevated IFN-γ compared with men (44, 48, 49). The more pronounced TLR7 activation in women likely serves as an explanation for the differing sex-specific patterns in inflammatory responses and clinical outcomes in the case of these pathogens.

#### 4.1.4 TLR9

In most studies, stimulation of human DCs with CpG, a TLR9 ligand, results in no sex bias in IFNα production (175, 177). However, a recent investigation using CpG-A indicates increased type I and II IFN expression in PBMCs obtained from blood samples from women (176).

When considering animal models, mouse cytomegalovirus (MCMV) infection leads to an upregulation of TLR9 expression in male immune cells compared with female cells. In MCMV-infected wild-type mice, males exhibit higher neutrophil and DC infiltration. However, in TLR9 KO mice, there are no sex differences following infection (179).

## 5 TLRs and sex hormones

Sex bias in TLR expression and TLR pathway–associated proteins have been progressively established. Whether the differential TLR expression in each sex is influenced by the hormonal environment or by sex chromosome–linked genes involved in the inflammatory response requires further investigation. As essential PRRs for a robust innate immune response and due to their significant involvement in infectious diseases, it is imperative to explore TLRs and their sex-specific variations. Sex hormones have been demonstrated to modulate the innate immune response. Namely, estrogens are known to be immunostimulators, whereas progesterone and androgens are considered immunosuppressors. The influence of these hormones on the expression of TLRs and TLR-mediated cytokine release may represent one of the primary factors contributing to sex-based disparities in immunity.

### 5.1 Estrogens and progesterone

#### 5.1.1 TLR2 and TLR4

Under conditions of bacterial stimulation, exposure to exogenous or naturally occurring estradiol tends to have an anti-inflammatory effect, resulting in decreased production of IL-1β, IL-6, TNF-α, and GM-CSF, while increasing the release of IL-10, IL-12, IL-23, and IL-27 (180). *In vitro*, human PBMCs stimulated with LPS and estradiol, show suppressed TNF-α production (159).

**Figure 3:**
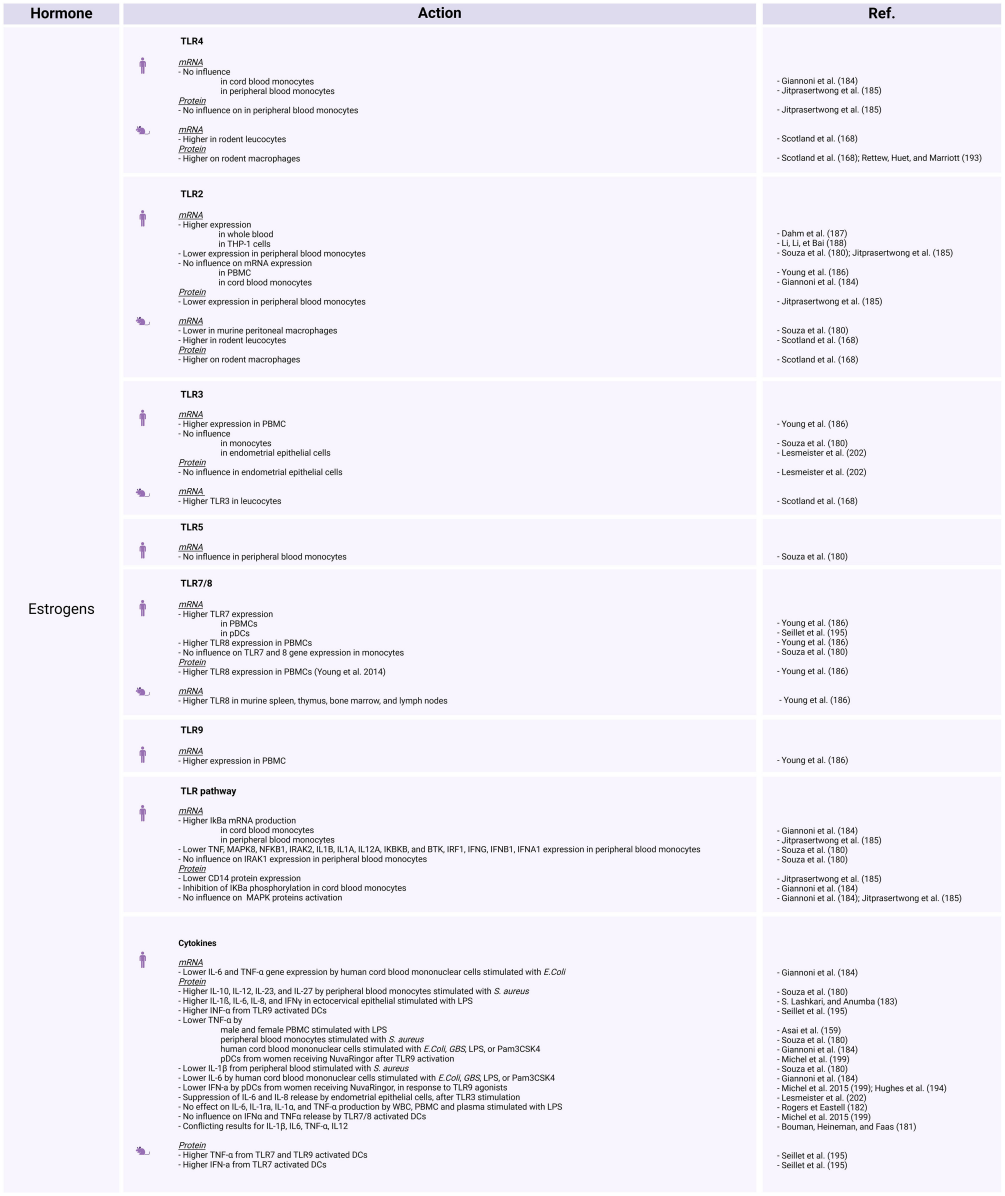
The influence of estrogens on TLR expression and activation in human and animal models. *Created with BioRender.com*.

**Figure 4:**
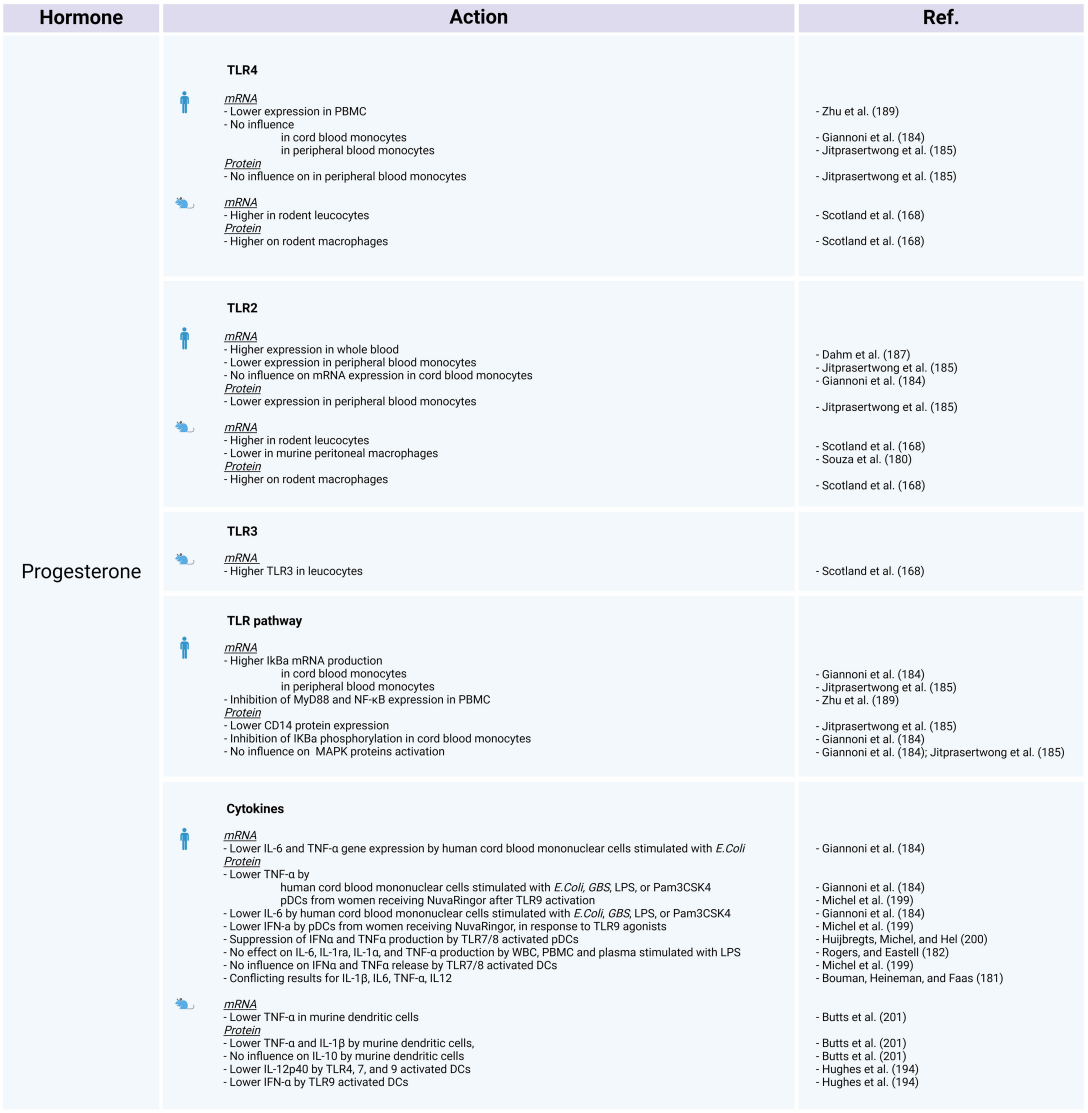
The influence of progesterone on TLR expression and activation in human and animal models. *Created with BioRender.com*.

On the other hand, a review from Bouman et al. (181), presents contentious evidence regarding the impact of estrogens and progesterone on IL-1β, IL-6, TNF-α, and IL-12 expression. Additionally, a study involving menopausal women who underwent hormone replacement therapy revealed no significant effects on IL-6, IL-1ra, IL-1α, and TNF-α production (182), while Another investigation focusing on human ectocervical epithelial cells demonstrated that following LPS stimulation, estradiol treatment increases the secretion of IL-1β, IL-6, IL-8, and IFN-γ compared with untreated cells (183). At first sight, the impact of estrogen and progesterone on the expression of inflammatory cytokines appears to be inconsistent among different studies (**Figures 3**, **4**).

A study investigating the impact of sex on stimulated human cord blood mononuclear cells, reveals that estradiol and progesterone decrease TNF and IL-6 protein and gene expression (184). The authors assessed *TLR2* and *TLR4* mRNA expression, as well as the protein levels of components within the NF-κB and MAPK pathways. Estradiol and progesterone do not influence *TLR2* or *TLR4* mRNA expression or the MAPK pathway, but do mediate cytokine release by inhibiting the phosphorylation of IκBα and increasing *NFKBIA* mRNA levels (184). The study concluded that the absence of phosphorylation prevents the ubiquitination and degradation of the IκBα protein, while the elevated mRNA levels suggest a potential increase in IκBα transcription. Under these circumstances, IκBα is unable to release the NF-κB p65 subunit, leading to inhibition of the NF-κB pathway (184). This finding could account for the observed difference in NF-κB dependent cytokine release regulation between women and men, with women showing more regulated release and men displaying higher levels of pro-inflammatory cytokines.

Jitprasertwong et al. (185) confirmed that exposure to estradiol and progesterone blocks the NF-κB pathway but does not affect TLR4 gene and protein expression or activation of MAPKs (phosphorylated forms of p38, ERK1/2, and JNK). The same authors also reported *TLR2* mRNA and protein downregulation, as well as reduced CD14 protein expression on stimulated human monocytes exposed to estradiol and progesterone (185).

Reduced *TLR2* mRNA expression was also observed after stimulation with *S. aureus* in male monocytes treated with 17β-estradiol and monocytes from women during their fertile period (180). However, conflicting findings arise again for these receptors; while some studies indicate no influence on *TLR2* mRNA (186), others demonstrate its upregulation in whole blood exposed to 17β-estradiol or progesterone (norethisterone), as well as in THP-1 cells treated with 17β-estradiol (187, 188). When female PBMCs are treated with progesterone, it results in the inhibition of both responsiveness and TLR4 expression (189).

Relative to other TLR pathway–related proteins, comparison of the gene expression profiles of women during their fertile period and men has revealed several noteworthy distinctions. In stimulated female monocytes TNF, MAPK8, and other genes are downregulated. Likewise, a comparison between male immune cells exposed to 17β-estradiol and their untreated but stimulated counterparts demonstrated that estradiol negatively regulates the *NFKB1*, *IRAK2*, *IL1B*, *IL1A*, *IL12A*, *IKBKB*, and *BTK* genes, but does not affect the *IRAK1* gene (180). Stimulated female PBMCs treated with progesterone show markedly reduced *MYD88* and *NFKB1* mRNA levels (189).

**Figure 5:**
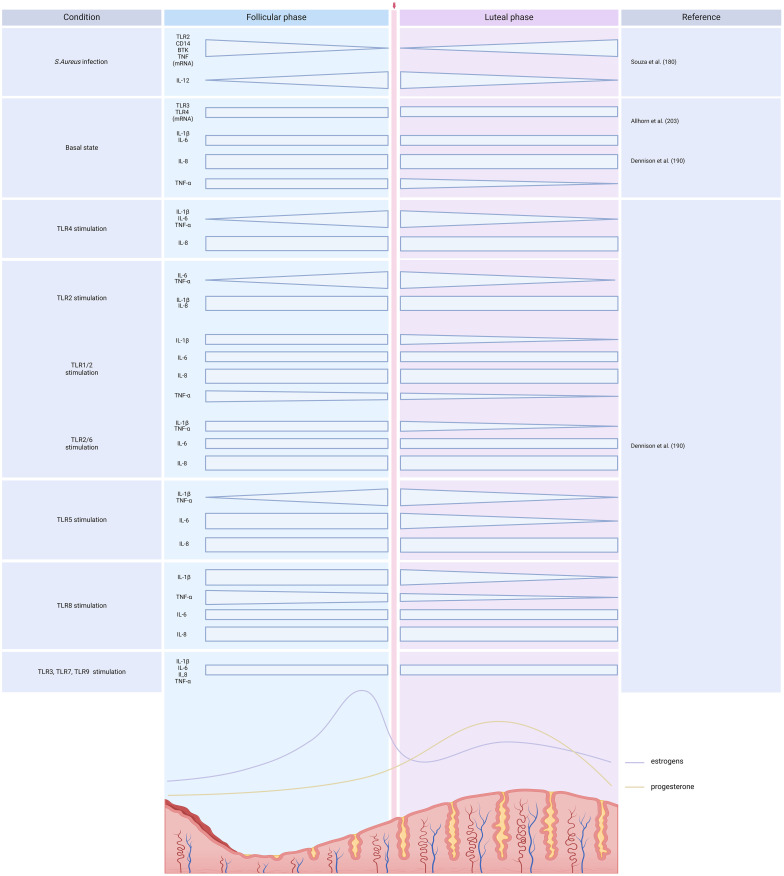
TLR expression and activation across the menstrual cycle. *Created with BioRender.com*.

To delve deeper into the influence of sex steroids on immune responses, several studies have examined TLR2 and TLR4 pathways modulation in women, across the menstrual cycle or during pregnancy (**Figure 5**). According to some researchers, female hormones impede relative *TLR2*, *CD14*, *BTK*, and *TNF* gene expression in human peripheral blood monocyte, while higher IL-12 levers are observed in the presence of these hormones (180). On the other hand, higher cytokine responsiveness to TLR2 activation is observed during follicular phase when estrogen is predominant, and then decreases during the luteal phase marked by progesterone presence (190). Some publications suggest that TLR4 expression remains consistent in women throughout the menstrual cycle (191), while others describe increased TLR4 responsiveness during the early luteal phase, which then decreases during the late luteal phase (190). According to Ziegler et al. (192), at the beginning of pregnancy, stimulation of TLR4 in PBMCs induces the release of TNF-α, which subsequently decreases as the pregnancy progresses.

Overall, the abovementioned studies have yield highly contrasting outcomes concerning the expression and activation of TLR2 and TLR4 following exposure to estrogens and progesterone (**Figures 3**-**5**). The unclear influence of these steroids challenges their role as the primary etiology of sex differences in TLR2- and TLR4-mediate inflammatory responses in humans.

In animal models, estradiol enhances the expression of TLR4 on rodent macrophages (193). In addition, ovariectomy reduces the expression of TLR2 and TLR4 in leukocytes and protein expression of TLR2 and TLR4 on macrophages (168). On the other hand, Souza et al. (180) reported that murine peritoneal macrophages from ovariectomized females express higher *TLR2* mRNA levels than wild-type females. Moreover, treatment of ovariectomized females or males with estradiol decreases TLR2 expression. In mouse plasmacytoid dendritic cells (pDCs), progesterone exposure decreases the IL-12p40 induction through TLR4 (194). These findings highlight the complex interplay between sex hormones and TLR expression in animals.

#### 5.1.2 TLR7 and TLR8

Estradiol increases TLR8 expression, both at the RNA and protein levels, in PBMCs from healthy premenopausal women. Moreover, it enhances *TLR7* gene expression in PBMCs from healthy premenopausal females and pDCs from postmenopausal women (186, 195). However, in monocytes, *TLR7* and *TLR8* gene expression does not vary between fertile women and men or between cells treated compared to those not treated with estradiol (**Figures 3**, **4**) (180).

The production of IFN-a, which relies on TLR7 activation, is reduced in PBMCs from women in the first trimester of pregnancy; however, there is an increase in stimulated pDCs during pregnancy (1, 192). Notably, the menstrual cycle does not affect TLR7 activation (190). In contrast, a decrease in responsiveness is described after TLR8 activation during the luteal phase (190). Remarkably, higher TLR7-induced IFN levels in pDCs from women compared with men (196) occur even before puberty (**Figures 3**–**5**) (197), prompting further discussion about the actual role of sex steroids in immune dimorphism and cytokine release.

Studies investigating the influence of hormonal contraception on TLR7/8 signaling have yielded mixed results. Some have described no significant difference in DC activation in women receiving depot medroxyprogesterone acetate (DMPA) or NuvaRing compared with those not using contraception (198, 199). Other studies suggest an inhibitory effect of medroxyprogesterone acetate on the production of IFN-α and TNF-α by TLR7–TLR8 activated pDCs (200). Interestingly, the presence of estrogen during the fertile period downregulates the *IRF1*, *IFNG*, *IFNB1*, and *IFNA1* genes in monocytes, all of which are involved in TLR7/8 signaling (180).

In mice exposed to estradiol, there is an increase in *TLR8* mRNA in various tissues, including the spleen, thymus, bone marrow, and lymph nodes (186). Mouse DCs exposed to estradiol display increased TNF-α release following TLR7 and TLR9 activation (195). Interestingly, ablation of the *ESR1* gene (which encodes estrogen receptor α) in DCs abolishes the TLR7-mediated IFN response (195). In murine DCs, pre-treatment with progesterone impairs the ability to release TNF-α and IL-1β, as *TNF* gene expression is also disturbed in a dose-dependent manner (201). On the contrary, progesterone exposure does not affect the production of IL-10 (201). However, progesterone exerts a negative influence on IL-12p40 induction through TLR 7 stimulation in mouse pDCs (**Figures 3**, **4**) (194).

#### 5.1.3 TLR9

Researchers have reported diverse effects of estrogens on TLR9. Higher *TLR9* gene expression is registered in PBMCs exposed to estradiol (186). In DCs, the presence of estradiol induces higher amounts of cytokine release upon TLR9 activation (195). Conversely, the use of hormonal contraception (such as DMPA or NuvaRing) or *in vitro* progesterone treatment reduce the capacity of pDCs to produce IFN-α, TNF-α, or IL-12p40 in response to TLR9 agonists (194, 199, 200). In rodents, DMPA hampers the production of IFN-α by pDCs following TLR9 activation or viral infection (**Figures 3**, **4**) (194).

#### 5.1.4 TLR3

There is limited information available on the influence of sex hormones on TLR3 expression and activation in humans. PBMCs treated with estradiol show enhanced *TLR3* mRNA expression (186), while there are no differences in monocytes (180). In endometrial epithelial cells, estradiol does not affect TLR3 gene or protein expression, but suppresses the release of IL-6 and IL-8 upon TLR3 stimulation with poly I:C. Lesmeister et al. (202) suggest that these results may be attributed to estradiol modulating the function rather than the quantity of this receptor. Nonetheless, no variations in TLR3-induced cytokines or *TLR3* mRNA expression have been identified throughout the menstrual cycle (190, 203). In mouse models, ovariectomy decreases TLR3 expression in leukocytes and TLR3 protein expression on macrophages (**Figures 3**–**5**) (168).

#### 5.1.5 TLR5

There is also scare literature regarding whether there is a TLR5 sex bias. In monocytes treated with 17-β estradiol, there are no differences in *TLR5* mRNA expression (180). Concerning TLR5-induced cytokines during the menstrual cycle, an increase in IL-1β and TNF-α release has been described from the follicular to the early luteal phase, while a decrease in IL-6 has been noted during the luteal phase (**Figures 3**–**5**) (190).

### 5.2 Androgens

In contrast to female sex hormones, there is very little information on the influence of androgens on the modulation of TLRs and their associated signaling pathways in human cells. Exposing PBMCs to testosterone does not affect the expression of TLR2, TLR3, TLR4, TLR7, TLR8, and TLR9 (186).

Given the clinical evidence discussed previously, which indicates that men have a more proinflammatory cytokine profile than women, it is intriguing to find that research on the impact of androgens on cytokines suggests a suppressive effect. In fact, there is a correlation between elevated concentrations of androgens and increased production IL-10 via TLR9 stimulation in male PBMCs (172). Observations in PBMCs and whole blood from patients with androgen deficiencies have revealed increased production of proinflammatory cytokines, specifically IL-1β and TNF-α, both at baseline and following exposure to LPS (204). Remarkably, exogenous testosterone administration negatively affects proinflammatory cytokine release (191). This inverse relationship between exogenous testosterone levels and TNF-α and IL-1β production has been confirmed in studies investigating diseases characterized by low androgen levels and hormone replacement therapy (205, 206).

In cultured human umbilical vein endothelial cells, exposure to testosterone inhibits the NF-κB DNA-binding activity (207). Correspondingly in umbilical cord serum, testosterone levels are negatively associated with TNF-α, while IL-10 is increased (208). These findings suggest that testosterone may mitigate the production of proinflammatory cytokines by downregulating NF-κB activity, although it does not appear to exert a direct influence on the expression of TLR genes or proteins in humans.

In animal models, testosterone upregulates IL-10 expression and reduces TNF production in LPS-treated murine macrophages (209). Similarly, in castrated rats, exogenous testosterone decreases the expression of TNF-α in a dose-dependent manner (210) within the prostate. In contrast to findings in human diseases, the absence of endogenous testosterone following orchidectomy, has been associated with an increased TLR4 expression on murine macrophages as well as the susceptibility to endotoxin shock. In contrast, control mice or those subjected to orchidectomy but subsequently treated with exogenous testosterone replacement exhibit reduced TLR4 expression and a corresponding decrease in endotoxin-induced severity scores (211).

Based on the insights gained from both animal and human studies, it appears that testosterone tends to have anti-inflammatory properties, in contrast to the proinflammatory patterns often observed in clinical settings in men. This intriguing paradox might be elucidated by examining potential variations in receptor kinetics or considering the role of hormones, which may not be as critical in underpinning sex-specific differences in immune responses as previously thought.

Indeed, according to the above evidence, estrogens, progesterone, and testosterone exert a discernible influence on the innate immune response, and, among other functions, the expression or function of TLRs. However, the emergence of sex-related inflammatory responses in prepubertal children, along with discernible TLR sex disparities prior to the onset of puberty, suggest an additional mechanistic layer at play, potentially involving genetic factors such as chromosomal genes (32, 197, 212, 213). Notably, genetic polymorphisms located on the X chromosome contribute to the immune sex bias, manifesting as differences in susceptibility to bacterial and viral infections between men and women (214).Therefore, determining the influence of sex chromosomes on TLR-mediated responses might enhance our understanding of the intricacies underlying the immune sex bias.

## 6 TLRs and the X chromosome

Women carry a pair of X chromosomes, while in men, the X chromosome is paired with the Y chromosome. The X chromosome harbors more than 1,000 genes, contrary to the Y chromosome, on which less than 100 genes have been identified. Several of these X-linked genes encode proteins integral to the functioning of the innate immune response (215–218). Female cells undergo random inactivation of the X chromosome, also referred to as silencing. This process leads to the aleatory expression of only one of the parental X chromosomes in each cell. The chromosome that will be inactivated is coated with the long noncoding RNA Xist, located on the X-inactivation center, which triggers a biological process resulting in chromosome condensation by heterochromatin modifications (2, 219–221).

Recent studies have described a more complicated procedure of X methylation in women. At least 23% of X-linked genes escape inactivation, inducing their overexpression. Thus, X-chromosome inactivation (XCI), designed to balance gene expression in women, may be incomplete, and some genes are expressed from both X chromosomes (2, 217, 222, 223). In addition to XCI, another mechanism leading to imbalanced gene expression in women is skewed XCI. In this case, X-chromosome silencing does not occur randomly; rather, one of the parental X chromosomes is preferentially expressed in nearly all cells (2, 217, 222, 223). Of note, the presence of only one X chromosome in men, in addition to the occurrence of skewed XCI in women, explain why the majority of pathologies related to X-linked genes predominantly affect boys and men. This imbalance in the expression of X-linked genes between men and women may contribute to immune sex differences and, particularly with respect to TLR disparities, may offer a more diversified immune response in women (2, 217, 222, 223).

**Figure 6:**
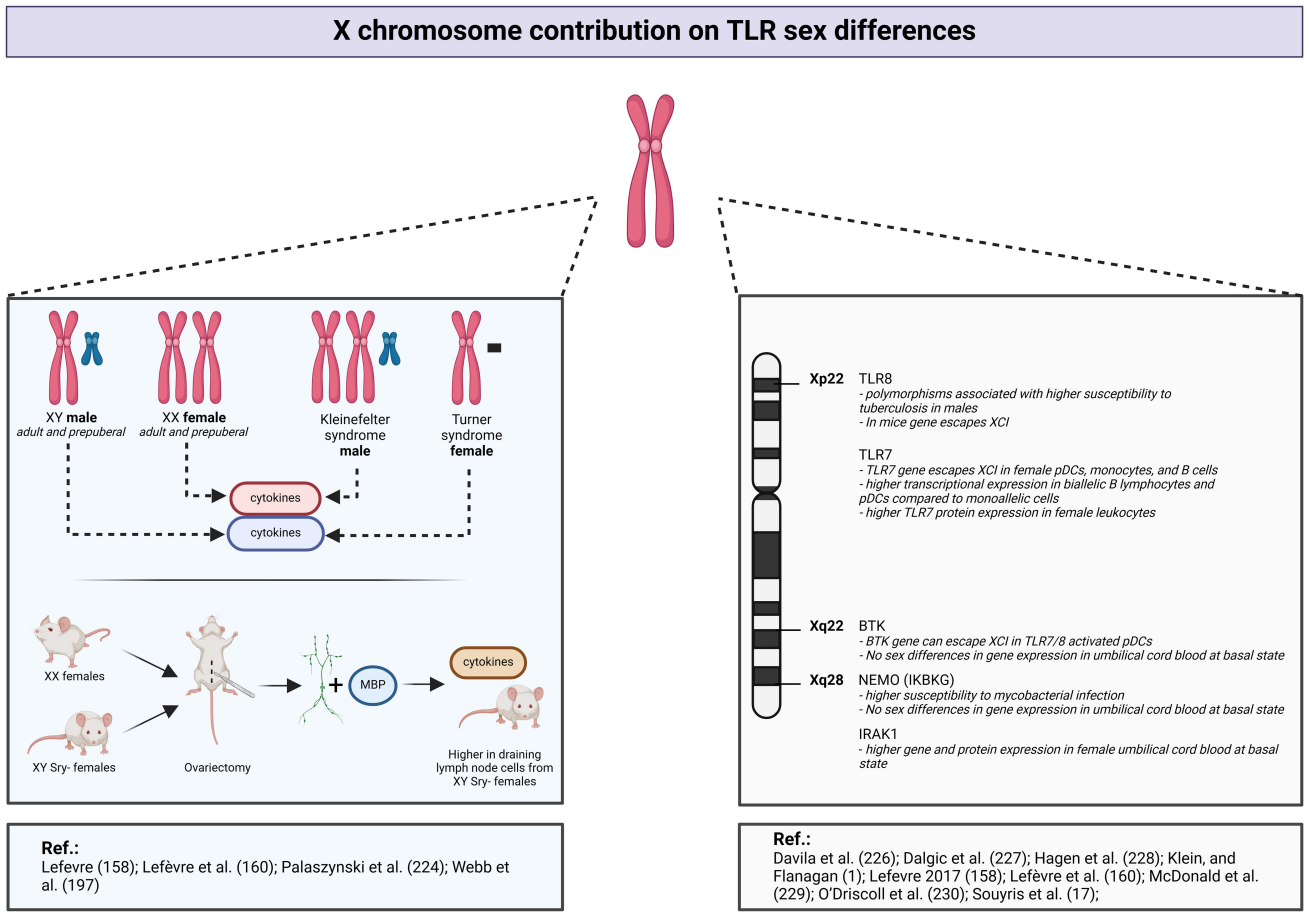
X chromosome contribution to TLR sex differences. *Created with BioRender.com*.

In one study, researchers assessed individuals with aneuploidies, comparing XY men and XX women, to evaluate the respective contribution of the X chromosome and sex hormones (**Figure 6**) (158). The authors measured the activation of some TLRs and found significant disparities in the innate immune responses between men and women. Indeed, whole blood stimulation, employing TLR2, TLR4, and TLR7/8 ligands, induces augmented release of proinflammatory cytokines in men (158). Individuals with Klinefelter syndrome, who exhibit a male phenotype despite the presence of an extra X chromosome, display a cytokine release profile similar to that of XX women (158). However, their estradiol levels do not significantly differ from those observed in women (158). These findings underscore the influential role of sex chromosomes over sex steroids. Moreover, the production of inflammatory cytokines in response to TLR ligands does not differ significantly according to the level of 17-β estradiol, reinforcing the less pronounced role of estrogens in these immune responses (160). Further investigation, conducted on purified monocytes, demonstrated lower levels of inflammatory cytokine production in women compared with men upon TLR activation (160).

An animal model employing XX female mice and XY-Sry deficient mice has provided additional information regarding sex-related differences in immune responses (**Figure 6**) (224). The *SRY* gene is a critical determinant in male fetal development. The absence of this gene leads to suppression of male differentiation, and thus the above mice are XY females. After ovariectomy in all groups of mice, the researchers collected draining lymph nodes cells and stimulated them with myelin basic protein (224). Despite the absence of gonadal hormonal influences and similar phenotypes, sex differences in proinflammatory cytokine release persist, depending on the number of X chromosomes (224).

Crucial components of the TLR-mediated innate immune response are under the genetic control of loci located on the X chromosome. Specifically, X-linked genes play a pivotal role in the regulation of TLR7, TLR8, as well as key constituents of the NF-κB pathway, including IRAK-1, NEMO, and BTK (**Figure 6**) (174, 216–218, 225).

Several studies have reported a relation between TLR8 polymorphisms and tuberculosis susceptibility, with a more pronounced effect observed in men (226, 227). These findings imply that mutations in the *TLR8* gene, located on the X chromosome, will have a greater impact in men who carry only one parental chromosome.

As discussed previously, TLR7 stimulation results in higher IFN production in women (196), even before the onset of puberty (197). Notably, according to a study including adults, prepubertal children, transgender men and women, and individuals with Turner syndrome, the IFN-1 production from TLR7-activated DCs is associated with the number of X chromosomes (197). More precisely, regardless of the sex steroid concentrations, the production of IFN production by pDCs following TLR7 stimulation is higher in subjects with a karyotype including two X chromosomes (prepubertal girls, adult women, and individuals assigned female at birth who identify as transgender) compared with subjects with a single X chromosome (prepubertal boys, adult men, individuals assigned male at birth who identify as transgender, and patients with Turner syndrome) (197). Transcriptional analyses conducted before and after vaccination in human subjects or following virus challenge in animal models show higher amounts of TLR-pathway and proinflammatory genes expression, such as TLR7 or NF-κB in female PBMCs and tissues (1).

Souyris et al. (174) and more recently Hagen et al. (228) have demonstrated that the *TLR7* gene, located on the X chromosome, escapes X inactivation in pDCs, monocytes, and B cells. This phenomenon leads in higher *TLR7* expression in women. Indeed, female biallelic B cells and pDCs express higher *TLR7* mRNA levels than monoallelic cells, and female leukocytes have greater TLR7 protein levels (174, 228). Similar pattern of TLR7 X-inactivation escape was observed in patients with Klinefelter syndrome, who possess an extra X chromosome (174). Thus, the differential expression and function of TLR7 showing a female predomination is mainly attributed to the X chromosome (**Figure 6**).

In mouse models, TLR8 also seems to escape from XCI; however, this phenomenon has not been proven in a human model (**Figure 6**) (229).

TLR1/2, TLR2/6, and TLR4 mainly activate the NF-κB pathway, which includes the X-related proteins IRAK-1, NEMO, and BTK. A recent study showed that the *BTK* gene can escape XCI, offering a potential explanation for certain sex-based variations in the TLR signaling pathway (**Figure 6**) (228). However, this observation was established in cells stimulated with a TLR7/8 agonist and not a TLR2 or TLR4 ligand. In neonatal PBMCs at the basal state, there are no sex-based differences in BTK or NEMO expression (**Figure 6**) (230). In contrast, IRAK1 gene and protein expression is higher in female PBMCs (**Figure 6**) (230). Nevertheless, this model was not evaluated under inflammatory conditions. Both clinical studies and *in vitro* experiments involving TLR2 and TLR4 ligands, show higher proinflammatory cytokine release in men (32, 160). However, the authors did not observe sex differences in TLR2 and TLR4 expression or the phosphorylated forms of NF-κB or MAPK-mediated pathway proteins, suggesting that cytokine sex bias is potentially induced at the transcriptional level (32, 160). These TLR2 and TLR4 signaling pathway differences have not yet been firmly established in *ex vivo* models.

The X chromosome also contains several microRNAs (miRNAs), which regulate protein synthesis by targeting specific mRNAs, thereby modulating gene expression through translational repression or enhancement. It has been suggested that X-linked miRNAs also undergo silencing and could contribute to the sex-specific immune response and TLR sex differences. Among miRNAs located on the X chromosome, interactions with TLRs have been described for miRNA-98, miRNA-223, and miRNA-105. miRNA-98, in synergy with let-7i, appears to regulate the TLR4 pathway, influencing SNAP3 and IL-10 expression. However, miRNA-98 itself is modulated by TLR4, as its expression is downregulated after LPS exposure (231–233). In contrast, miRNA-223 exhibits upregulation in response to TLR4 signaling and is implicated by modulation of neutrophil differentiation from myeloid precursors (233, 234). miRNA-105 has a negative regulatory effect on *TLR2* mRNA in human gingival keratinocytes: Higher levels are associated with oral keratinocytes from patients exhibiting a diminished TLR2 response and cytokine production (235).

Although research concerning the role of the X chromosome in TLR-related sex differences has been relatively limited compared with the extensive examination of sex steroids, emerging findings strongly emphasize the pivotal involvement of X-linked genes. The intricate influence of XCI mechanisms on gene expression introduces a source of diversity in the women’s immune response. The imbalanced expression of X-linked genes between men and women carries substantial implications for shaping immune responses and is implied to underlie the well-documented phenomenon of immune sex differences.

## 7 Conclusion and future perspectives

TLR expression is one variable that influences the sex-related innate immune response. In this review, we have summarized the TLR sex bias and have attempted to identify, based on the literature, the exact etiology. Studies concerning sex bias in TLR mRNA and protein expression have mostly concentrated on TLR2, TLR4, TLR7, and TLR8, as they are highly implicated in infectious diseases. Sex steroids influence the innate immune response and, among other functions, the expression and function of TLRs. Nonetheless, the exact effects of sex hormones on TLR4 and TLR7 remain ambiguous. On the other hand in studies with postmenopausal patients receiving a hormone replacement therapy, the cytokine proinflammatory response is not affected, suggesting an additional mechanistic layer at play, potentially involving genetic factors (32, 182, 197, 212, 213). Genetic polymorphisms located on the X chromosome contribute to the immune sex bias, manifesting as differences in susceptibility to bacterial and viral infections between men and women (214). In light of these findings, we strongly recommend further investigation of genes located on the sex chromosomes to understand sex disparities in the immune system and response.

As described above, some X chromosome genes are methylated and thus escape inactivation in women. This phenomenon has been confirmed for TLR7, an X-related protein, in pDCs, monocytes, and B cells, resulting in higher expression levels of this receptor in women. Based on these observations, we should also evaluate the expression of other X-linked proteins involved in the TLR pathways.

There are divergent responses regarding the sex-related expression of TLR2 and TLR4. However, their activation through ligands mainly shows an enhanced response in men, as denoted by cytokine release. The variability in these results demands further investigation into these receptors in humans to determine whether there are sex-dependent differences in TLR2 and TLR4 expression. If there are no sex differences, and considering the uncertain impact of sex steroids, attention must be drawn to proteins of the pathways that induce cytokine release. Notably, some of the NF-κB pathway proteins such as IRAK-1, NEMO, and BTK are encoded by genes located on the X chromosome (216–218). Findings regarding the contribution of X-linked genes to TLR4 sex differences have been extrapolated from studies focusing on NF-κB-mediated cytokine release. Additional research is needed to explore IRAK-1, NEMO, and BTK gene and protein expression, as well as the phosphorylation of downstream proteins in the pathway, such as NF-κB, p38, and ERK. This approach should clarify the mechanisms underlying sex differences from TLR activation to cytokine release.

The compelling engagement of TLR2 and TLR4, resembling their roles in bacterial infections, in cases of viral infections, characterized by a significant influx of neutrophils, warrants further exploration. The involvement of PMNs in TLR pathway responses has already been suggested in studies on TLR2 and TLR4 (100, 101). The dynamic interplay between neutrophils and TLRs should be subjected to close scrutiny, with a particular focus on unravelling the implications of this interaction in the context of sexual dimorphism.

The X chromosome also harbors miRNAs that could also escape XCI inactivation and modulate TLR expression. While some research has explored the relationship between miRNAs and TLRs, the question of causality remains. Indeed, there is more robust evidence that TLRs regulate miRNAs than for the reverse situation (236). Further investigation in this area is warranted.

Most of the information about TLR sex differences has been derived from animal models. However, these data are a poor reflection of human biology (237). Furthermore, different miRNAs have been described in mice and humans, drawing attention to the thoroughness required in this type of research. The human X chromosome encompasses 76 miRNAs and the mouse X chromosome contains 65, and there are only 37 miRNAs common to both species (233). Hence, while animal research undoubtedly contributes to scientific progress, subsequent investigations involving humans are essential to validate any findings.

Moreover, in humans, isolated cells do not adequately represent the complexity of whole blood (238–240). Isolated cell populations lack the necessary context of cell communication and the surrounding environment, which can lead to unintended activation, stability issues, and biased results. Whole blood studies offer a more comprehensive analysis of immune function. Thus, research on TLRs should integrate insights from animal models and be conducted in human whole blood to establish a more physiologically relevant model.

Uncovering the cell signaling pathways underpinning the inflammatory distinctions between women and men holds the promise of identifying specific prognostic markers for individuals with inflammatory diseases or acute infections. It may also reveal new therapeutic targets to modulate the inflammatory response based on the patient’s sex. Accordingly, gaining a deeper understanding of TLR mechanisms involved in acute inflammatory processes, such as sepsis, could prove instrumental in mitigating tissue damage and, thus, be of great benefit for patients.

To summarize, the emergence of sex-related inflammatory responses in prepubertal children, along with discernible TLR sex disparities prior to the onset of puberty, provides ample support for the assertion that genes situated on the X chromosome could be responsible for some of the differences between men and women in TLR expression and activation. Future clinical trials should identify the fundamental mechanisms of the inflammatory sex differences through TLRs. Research on immune sex differences might offer novel prognostic and therapeutic targets that will help advance personalized medicine in inflammatory diseases.

## Author contributions

AP: Writing – original draft, Writing – review & editing. GC: Writing – review & editing. FC: Writing – review & editing. NL: Writing – review & editing.
